# Variations in MHC class I antigen presentation and immunopeptidome selection pathways

**DOI:** 10.12688/f1000research.26935.1

**Published:** 2020-09-28

**Authors:** Anita J. Zaitouna, Amanpreet Kaur, Malini Raghavan

**Affiliations:** 1 Department of Microbiology and Immunology, Michigan Medicine, University of Michigan, Ann Arbor, MI, USA

**Keywords:** MHC class I, HLA class I

## Abstract

Major histocompatibility class I (MHC-I) proteins mediate immunosurveillance against pathogens and cancers by presenting antigenic or mutated peptides to antigen receptors of CD8+ T cells and by engaging receptors of natural killer (NK) cells. In humans, MHC-I molecules are highly polymorphic. MHC-I variations permit the display of thousands of distinct peptides at the cell surface. Recent mass spectrometric studies have revealed unique and shared characteristics of the peptidomes of individual MHC-I variants. The cell surface expression of MHC-I–peptide complexes requires the functions of many intracellular assembly factors, including the transporter associated with antigen presentation (TAP), tapasin, calreticulin, ERp57, TAP-binding protein related (TAPBPR), endoplasmic reticulum aminopeptidases (ERAPs), and the proteasomes. Recent studies provide important insights into the structural features of these factors that govern MHC-I assembly as well as the mechanisms underlying peptide exchange. Conformational sensing of MHC-I molecules mediates the quality control of intracellular MHC-I assembly and contributes to immune recognition by CD8 at the cell surface. Recent studies also show that several MHC-I variants can follow unconventional assembly routes to the cell surface, conferring selective immune advantages that can be exploited for immunotherapy.

## Introduction

**Figure 1.**
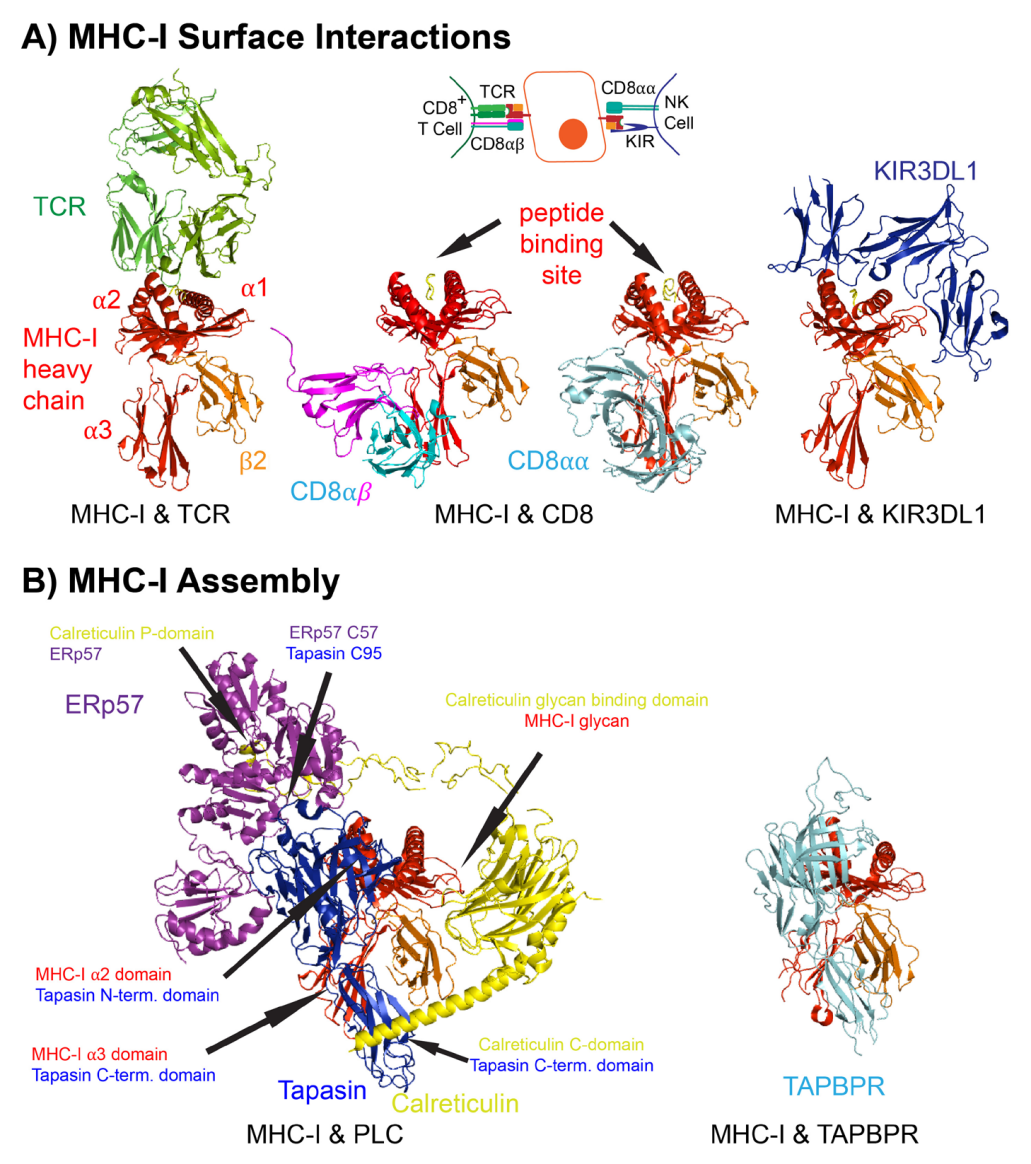
Major histocompatibility class I (MHC-I) surface interactions and assembly. ( **A**) Crystal structure and cartoon representation of MHC-I (red: heavy chain, orange: β2m, yellow: peptide)/TCR (green) (PDB 5C07 ^3^) on CD8 ^+^ T cells, MHC-I/CD8 co-receptor (cyan and magenta) (PDB 3DMM ^4^ and PDB 3QZW ^5^), or MHC-I/KIR3DL1 (blue) on natural killer (NK) cells (PDB 5B38 ^6^). ( **B**) Cryo-EM structure of MHC-I in the PLC (yellow: calreticulin, blue: tapasin, purple: ERp57) (PDB 6ENY ^7^ adapted from data freely accessible at: https://www.rcsb.org/structure/6ENY) or with TAPBPR (cyan) (PDB 5WER ^8^) in the peptide-deficient form. Arrows highlight interactions between tapasin and MHC-I, calreticulin and the MHC-I glycan, calreticulin and tapasin, calreticulin and ERp57, and tapasin and Erp57. β2m, beta2-microglobulin; KIR, killer cell immunoglobulin-like receptor; PLC, peptide loading complex; TAPBPR, transporter associated with antigen presentation-binding protein related; TCR, T-cell receptor.

Major histocompatibility class I (MHC-I) proteins are expressed on the cell surface of nucleated cells and serve critical functions in the immune response by mediating the activation of CD8 ^+^ T cells and regulating the activity of natural killer (NK) cells. MHC-I molecules form trimeric complexes that consist of a heavy chain, a light chain (beta2-microglobulin, or β2m), and peptide. T-cell receptors of CD8 ^+^ T cells and the CD8 co-receptors of the same cell engage the membrane-distal peptide-binding domain and the membrane-proximal domains, respectively, of individual peptide–MHC-I molecules, providing the initiating signal for CD8 ^+^ T-cell activation (reviewed in 1) ( Figure 1A). Various NK cell receptors can bind to specific MHC-I molecules to inhibit or initiate NK cell activation (reviewed in 1). The CD8 co-receptor is also expressed on NK cells. Whereas the CD8αβ heterodimer is expressed on CD8 ^+^ T cells, a subset of NK cells expresses the CD8αα homodimer ( Figure 1A). Recent studies show that KIR3DL1, an NK cell receptor for human MHC-I, uses the CD8αα homodimer as a co-receptor ^2^. Thus, CD8 functions as an MHC-I engaging co-receptor, not just for T cells but also for NK cells.

Since MHC-I molecules are critical ligands for receptors of both T cells and NK cells, their assembly and expression are subject to elaborate cellular quality control. The MHC-I complex is assembled in the endoplasmic reticulum (ER), travels to the Golgi apparatus, and follows the secretory pathway to reach the cell surface ^9, 10^. Assembly in the ER occurs with the help of the peptide loading complex (PLC), a large macromolecular assembly comprising TAP subunits TAP1 and TAP2, tapasin, ERp57, and calreticulin in addition to MHC-I heavy chain and β2m ^9^. Apart from the PLC proteins, ERAP variants ERAP1 and ERAP2 and TAPBPR are important players in peptide trimming and peptide quality control, respectively.

In humans, three sets of classical and non-classical MHC-I genes as well as several non-classic MHC-I genes encode the heavy chains of MHC-I proteins. The classical MHC-I genes are the human leukocyte antigen class I (HLA-I) genes *HLA-A*, *HLA-B*, and *HLA-C*. Each gene is polymorphic with multiple allelic variations, and more than 19,000 alleles were listed for classical *HLA-I* genes on the IPD-IMGT/HLA Database as of May 2020 ^11^. Allelic variants of individual genes frequently occur as groups of co-inherited alleles called haplotypes ^12^, which are jointly implicated in various disease susceptibilities ^13^. The non-classical MHC-I genes encode *HLA-E*, *HLA-F*, *HLA-G*, *cluster of differentiation 1 (CD1)*, and *MHC-related protein 1 (MR1)*. These genes display low allelic polymorphisms and engage various immune receptors to activate or inhibit immunity ^14^. A number of recent studies have addressed the question of how classical MHC-I polymorphisms influence the assembly, conformation, and expression of individual human MHC-I variants, the impact of polymorphisms on the peptide repertoires, and the functional consequences for immunity, topics that are the focus of this review article.

## Peptidomes of HLA-I molecules and the prevalence of spliced and post-translationally modified peptides

**Figure 2.**
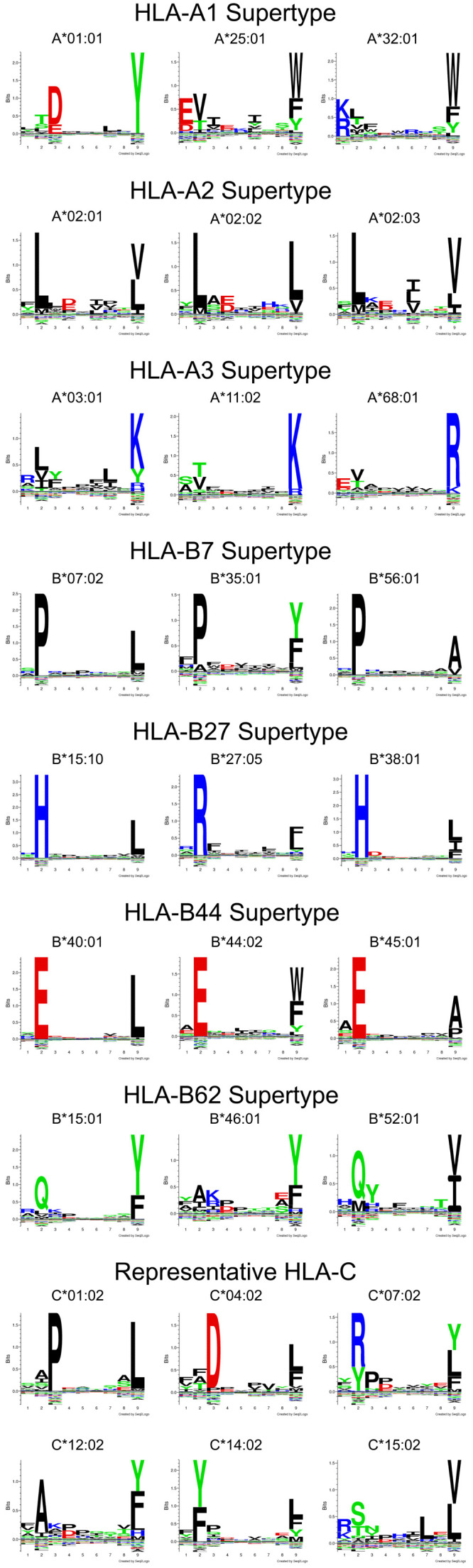
Binding motifs of 9-mer peptides bound to select HLA-A, HLA-B, and HLA-C. Seq2Logo motifs of eluted 9-mer peptides that bind to the specified HLA-A, HLA-B, and HLA-C allotypes grouped by supertype for HLA-A and HLA-B. All motifs are derived on the basis of analysis of eluted peptide sets ^19^ (adapted from data freely accessible at: ftp://massive.ucsd.edu/MSV000084172/). Within a given supertype ^32^, the peptide motifs are similar. Many HLA-A allotypes lack the strong P2 restriction generally seen in HLA-B and HLA-C. The HLA-B*07 allotype binds to peptides that are disfavored by transporter associated with antigen presentation (TAP), and allotypes belonging to this supertype are expressed at low levels on the surface of lymphocytes but at higher levels on TAP-deficient cells ^33^. HLA, human leukocyte antigen.

The heavy chains of MHC-I molecules contain a peptide-binding site ( Figure 1), which is also the site of amino acid variations that define MHC-I polymorphisms, resulting in variable peptide-binding specificities ( Figure 2) ^15^. Immunoaffinity purification in conjunction with mass spectrometric (MS) studies using data-independent acquisition (DIA) MS approaches has allowed for the identification of thousands of peptides associated with individual HLA-I variants (designated the peptidome) ^16^. Many of the MS studies have used HLA-I null cells that are engineered to express single HLA-I molecules (termed monoallelic), which are affinity-purified, followed by peptide elution and sequencing by liquid chromatography tandem MS (LC-MS/MS) approaches ^17– 19^. The monoallelic approach is advantageous over other methods in that the isolated peptidome can be attributed to a specific HLA-I without the need for prediction algorithms to assign peptides to a given HLA-I. A pan HLA-I antibody (W6/32) ^20^ can be used for immunoisolating most HLA-I proteins. On the other hand, prediction algorithms are typically required for peptide/HLA assignments when primary cells are used, which typically express six HLA-I molecules, two each of HLA-A, HLA-B, and HLA-C. Alternatively, immunoaffinity purification can be modified to target specific MHC-I molecules within primary tissues and cells via the use of antibodies specific to a small subset of MHC-I. Owing to the large diversity and close relatedness of many MHC-I molecules, few such HLA-I allotype-specific antibodies exist, and careful specificity controls must be performed prior to their use. The rather long duration of the affinity purification process is expected to result in the capture of only a subset of peptides displayed on the cell surface, especially those with high affinity and abundance ^21, 22^. Additionally, other parameters, including chemical properties of individual peptides, which have to be able to bind a C18 column and be ionizable for successful MS-based detection, can skew peptide detection ^23^. Nonetheless, the approach has resulted in the generation of datasets containing the identities of large numbers of actual HLA-I–bound peptides, expanded the list of alleles for which peptidome datasets are available, helped improve predictive algorithms for peptide binding to HLA-I, and confirmed that a single HLA-I displays thousands of cell surface peptides ^17, 19^. A recent study exploited the monoallelic approach to define the peptidomes of 95 HLA-I proteins ^19^. The resulting datasets have confirmed that peptide-binding motifs and sub-motifs are shared across HLA-I molecules that bear similarities in their peptide-binding sites ( Figure 2, individual supertypes) and that 9-mer peptides are dominant among the majority of tested allotypes (except for HLA-B*18:01 and HLA-B*52:01). The peptidome data have been combined with transcript abundance and peptide processing data to improve the available tools used for the prediction of peptides binding to a given HLA-I ^19^.

The proteasomes are the major cellular proteolytic system to generate peptides from precursor protein for MHC-I binding ^24^. In addition to conventional proteolytic activities, proteasomes are known to be able to catalyze transpeptidation reactions to generate spliced epitopes comprising non-contiguous segments from precursor proteins ^25, 26^. Recent studies have attempted to quantify the fraction of HLA-I ligandomes that correspond to spliced epitopes, both cis-spliced (spliced peptides originating from the same precursor) and trans-spliced (spliced peptides originating from different precursors) ^18, 27, 28^. The validity of some of the approaches used for quantifying the spliced peptide fraction remains somewhat controversial ^28^; to date, only a small number of the possible spliced peptides have been confirmed as products of proteasomal splicing or directly validated as spliced epitopes using other approaches. Thus, although it is clear that spliced peptides exist and function as T-cell epitopes in the immune response ^25, 26^, further studies are required to understand how frequently spliced epitopes might contribute to T-cell responses. Besides splicing, post-translational modifications of MHC-I epitopes further expand the peptide repertoires of presenting cells ^29^. Specific forms of glycopeptides have also been identified in leukemia patients that can activate cytotoxic T-cell responses ^30^.

## The classical MHC-I assembly pathway and its dysfunction in disease

In the MHC-I assembly pathway, peptides are generated in the cytosol by the proteasome. A fraction of the peptides are brought into the ER via TAP in an ATP-driven process ^31^. The PLC brings into close proximity many components for both stabilizing nascent MHC-I molecules and facilitating peptide binding to MHC-I heterodimers ^9^. A cryogenic electron microscopy (cryo-EM) structure of an entire PLC was recently solved at a resolution of 5.8 Å for most components (PDB 6ENY; Figure 1B) ^7^. Reconstruction of the low-resolution map was accomplished by superposition of higher-resolution crystal structures. The PLC comprises TAP1, TAP2, and two molecules each of tapasin, peptide-free MHC-I, and calreticulin–ERp57. Of these components, TAP1, TAP2, and tapasin are encoded within the MHC gene complex along with MHC-I heavy chains and function as dedicated assembly factors. Additionally, the PLC comprises two generic co-chaperones of the ER: the glycoprotein-specific chaperone calreticulin and the thiol oxidoreductase ERp57 ^9^. In the PLC, peptide-deficient MHC-I interacts with tapasin at two sites. The first site involves the interaction of membrane-proximal C-terminal domain of tapasin with the MHC-I α _3_ domain and β2m interface, close to the CD8-binding region of MHC-I. The second site involves the interaction of membrane-distal N-terminal domain of tapasin and MHC-I peptide-binding domain at the α _2–1_ region of MHC-I ( Figure 1B). The additional incorporation of the calreticulin–ERp57 complex into PLC stabilizes the tapasin–MHC interaction via two associations within the PLC: one between the N86-linked glycan of MHC-I and the glycan-binding site of calreticulin and other one formed by a disulfide-linked conjugate between C95 of tapasin and C57 of ERp57. The peptide-deficient form of MHC-I is generally unstable and is the form recruited to the PLC. The structure of the PLC illustrates how a peptide-deficient MHC-I becomes stabilized by multi-pronged interactions with dedicated and generic chaperones ^7^. Peptide binding causes the release of peptide-bound MHC-I from the PLC, allowing forward trafficking into the Golgi apparatus.

MHC-I molecules are critical for effective immunity, and a functional PLC, in turn, is critical for effective MHC-I assembly. Many viruses and cancers are known to disrupt the functions of components of the PLC to evade immune recognition (reviewed in 34– 36). In particular, herpesviruses encode many proteins dedicated to the inhibition of TAP expression or function ^34^. A recent study shows that tapasin becomes a degradation target in cells infected with molluscum contagiosum virus, thereby negatively affecting MHC-I assembly ^37^. Somatic mutations of calreticulin have recently been reported in a subset of myeloproliferative neoplasms (MPNs). Most of these mutations are mapped to exon 9 of the calreticulin gene and alter the protein reading frame such that the C-terminus of mutant calreticulin becomes enriched in basic amino acids, in contrast to the acidic C-terminus of the wild-type protein ^38, 39^. The two most common mutations are 52–base pair deletion (type 1) and 5–base pair insertion (type 2) mutations ^39^. These mutant proteins are known to be secreted since they lack the KDEL retention sequence (reviewed in 40). The significance of calreticulin in MHC-I antigen presentation was demonstrated in earlier studies that showed downregulation of surface MHC-I and impaired antigen presentation in calreticulin-deficient cells ^41, 42^. Mutants of glycan binding residues of calreticulin show reduced incorporation of calreticulin and MHC-I into PLC and reduced MHC-I assembly and cell surface expression ^42^. Interestingly, the surface expression of MHC-I is lower in cells expressing MPN mutants of calreticulin compared with those expressing wild-type protein ^43^, despite an intact glycan-binding site on the mutants. The weaker interactions of mutant calreticulin proteins within the PLC and reduced MHC-I surface expression compared with wild-type calreticulin are observed even for recombinant MPN calreticulin mutants engineered to contain a C-terminal KDEL sequence. Notably, within the PLC structure, the C-terminal helix of calreticulin points toward the ER-luminal membrane leaflet and lies close to the C-terminal immunoglobulin-like domain of tapasin ^7^ ( Figure 1B), and previous studies also show that various (non-MPN) calreticulin mutants negatively influence cellular steady-state levels of tapasin ^42^. Loss of interaction between the C-terminal helix of calreticulin and tapasin in the context of mutated calreticulin can be postulated to account for the inability of cells expressing MPN calreticulin mutants to restore the MHC-I surface expression to normal levels even when a KDEL sequence is added to mutant calreticulin proteins ^43^. These and other recent findings indicate a role for MPN mutant calreticulin-mediated immunosuppression in tumor development and progression ^43, 44^ in addition to the more direct cell-transforming potential of the calreticulin mutants ^40^.

## Some HLA-I variants can acquire peptides via TAP- and tapasin-independent pathways, which may confer immune advantages

Some HLA-I allotypes are known to be able to load peptides and reach the cell surface even in the absence of a functional TAP or tapasin ^33, 45– 49^. Recent studies involving many of the frequent North American HLA-B allotypes revealed a range of abilities to become expressed under TAP- or tapasin-deficient conditions ^33, 49^. These findings suggest the widespread prevalence of allotypes for which the conventional assembly routes are non-essential for measurable surface expression. Considerable attention in the field has been focused on the mechanisms that govern tapasin- and TAP-independent cell surface expression. Generally, but not in every case, the degrees of TAP-independent and tapasin-independent expression correlate with each other and with the intrinsic stability of a peptide-deficient HLA-B, which allows efficient assembly independently of the assembly factors ^33, 49^. Additionally, some allotypes have more efficient HLA-I assembly and surface expression under TAP-deficient conditions than others because those HLA-I molecules bind peptides that are better represented within signal sequences or protein transmembrane domains, allowing higher levels of peptide access in the ER lumen independently of TAP ^33, 45^. Recent studies involving the analysis of MHC-I peptidomes from spleen cells of TAP-deficient mice found that the peptidome was enriched in signal sequence–derived peptides as well as those derived from precursors in the extracellular space ^50^. Notably, endoglycosidase H–resistant forms of HLA-B are detectable on the cell surface under TAP-deficient conditions, indicating a role for unconventional secretory pathways in trafficking of HLA-B from the ER to the cell surface in TAP-deficient cells ^33^. Although the nature of these pathways remains to be elucidated, it is possible that this mode of trafficking allows for peptide loading of endocytosed extracellular antigens within endolysosomal compartments. It is also noteworthy that some HLA-B allotypes that display higher levels of expression on TAP-deficient cells bind peptides with a proline residue at the P2 position, which are disfavored for transport by TAP ^51^, and such HLA-B allotypes are expressed at low levels on the surface of primary lymphocytes ^52^. It is possible that such allotypes have evolved to enable some level of antigen presentation under pathogenic conditions in which the normal TAP-dependent pathway becomes blocked.

For some alleles, the peptide repertoire size of MHC-I molecules has been discussed as being correlated to tapasin-independent and TAP-independent expression levels ^53, 54^. These suggestions are based in part on studies that showed that chicken MHC-I molecules had varying promiscuities of peptide binding (peptide repertoire sizes) that inversely correlated with surface MHC-I expression levels ^53^. By extension, low-expressing human MHC-I allotypes have been suggested to be promiscuous peptide binders (based on earlier peptide repertoire predictions ^55, 56^) and were noted to be tapasin-independent ^53^. More detailed studies showed that, in primary human cells, the cell surface expression patterns of MHC-I molecules were complex and both allele- and cell type-dependent. As noted above, expression variations are determined at least in part by the match between the binding specificities of TAP and the MHC-I allotype ^52^. Furthermore, there are at least two major and distinct determinants of a larger peptide repertoire size for a given MHC-I. These are (i) the intrinsic structure of the peptide-binding site that can result in a broader peptide repertoire (examples such as A*25:01, B*15:01, and C*15:02 are shown in Figure 2) and (ii) high intrinsic stabilities of the peptide-deficient forms and high efficiencies of peptide loading, which can result in the presentation of suboptimal epitopes under suboptimal assembly conditions relevant to many infections and cancers. Many TAP/tapasin-independent allotypes are expected to be capable of binding and presenting suboptimal epitopes on the basis of their high intrinsic stabilities and efficiencies of peptide loading, but such expanded peptide repertoires may not be captured by conventional LC-MS/MS methods and derived predictive tools, which, as discussed above ^22^, are best able to identify high-affinity epitopes. Thus, immunological approaches in addition to predictive and MS approaches are required to define and compare the full peptide repertoire sizes of MHC-I allotypes.

Residue 114 and 116 ^47– 49, 57– 59^ as well as other amino acid positions (residues 97 ^60^, 147 ^61^, and 156 ^60, 62^) are known to be important determinants of tapasin dependence of MHC-I. Molecular dynamics (MD) simulations have been performed of HLA-B allotypes that differ by a single amino acid at position 116 and in their tapasin dependencies ^61, 63– 65^. With some HLA-B pairs, these studies indicate a greater structural stability of the F pocket (near the peptide C-terminus) of tapasin-independent allotypes in their peptide-deficient forms ^64– 66^ as well as greater α _1_ helix flexibility of peptide-bound forms of tapasin-dependent allotypes ^63^. In contrast, MD studies with a set of closely related HLA-B pairs found that the tapasin-independent allotype was more dynamic when peptide was absent compared with the tapasin-dependent allotype ^61^. Although further studies are needed to compare the dynamics of different sets of tapasin-dependent or tapasin-independent allotypes under similar experimental conditions, these different results with the distinct allelic pairs raise the possibility that distinct sets of conformational variations can influence the degree of tapasin independence. Nuclear magnetic resonance (NMR) studies have shown chemical shift variations in β2m residues at the heavy chain–β2m interface between a pair of tapasin-dependent or tapasin-independent allotypes that differ only at residue 116 ^67^. Such variations can be indicative of potential differences in the stabilities of specific β2m–heavy chain complexes in the absence of peptides, which in turn could render the PLC less critical for peptide loading for some allotypes. Remarkably, not only do tapasin and CD8 share a binding site on MHC-I ( Figure 1) but CD8, like tapasin, has higher apparent affinity for the peptide-deficient form of MHC-I. Peptide-deficient forms of MHC-I are induced on the cell surface under some conditions, and once there, these forms can engage CD8 at the immune synapse and enhance antigen-specific immune responses ^68^. Thus, MHC-I conformational sensing is used by both ER assembly factors and cell surface receptors for MHC-I.

The identification of TAP-independent routes of peptide transport to the ER has generated interest in the development of strategies to exploit TAP downregulation in cancer for enhancing anti-tumor immunity. In cancers, the proteolytic products of mutated proteins (termed neoantigens) can be assembled with MHC-I for presentation to CD8 ^+^ T cells, resulting in the activation of protective anti-tumor CD8 ^+^ T-cell responses ^69^. Such presentation is indispensable for immune control of cancer and for the success of immunotherapy-based cancer treatments. Additionally, TAP-independent routes of peptide transport in tumors with TAP downregulation may allow presentation of MHC-I epitopes called TEIPPs (T-cell epitopes associated with impaired peptide processing) which are derived from non-mutated self-proteins that are not presented by TAP-proficient cells ^70^. Several such HLA-A*02:01-restricted neoantigens that are potential candidates for cancer immunotherapy have been identified. CD8 ^+^ T cells specific to these epitopes are present in healthy donors and are not affected by tolerance mechanisms ^71^. T cells against an epitope derived from the leader sequence of LDL receptor-associated protein 1 (LRPAP1) are shown to recognize TAP-deficient tumor cells of different histological origins, but not healthy cells ^71^. Based on these findings, recent studies focused on the evaluation of a therapeutic model that involved the use of targeted knockdown of TAP in tumor cells to enhance the efficacy of conventionally used checkpoint inhibitors and to test the potential of TEIPP-based peptide vaccines in cancer therapy ^72, 73^. The success of these strategies in the clinic, though promising, is bound to be affected by several factors, including the HLA-I genotype of the patients, identity of specific antigens being tested, and the type of cancer.

## TAPBPR recognizes peptide-deficient and peptide-filled HLA-I variants

TAPBPR is structurally related to tapasin ^74– 77^ ( Figure 1B), but unlike tapasin, TAPBPR does not incorporate into the PLC ^78^. It has been found in both the ER and the Golgi apparatus ^78^. Similar to tapasin ^79^, TAPBPR preferentially binds the peptide-deficient form of MHC-I, and binding of selected peptides to MHC-I can destabilize its association with TAPBPR ^80, 81^. However, complexes between peptide-bound MHC-I and TAPBPR have been detected for some MHC-I molecules ^76, 77, 82^. The TAPBPR–MHC-I interaction appears to be higher-affinity than tapasin–MHC-I complexes, as stable complexes of MHC-I with TAPBPR but not tapasin are detected by gel filtration chromatography and by analytical ultracentrifugation for the same MHC-I allotype ^81^. Thus, it has been possible to study the structure and dynamics of the TAPBPR and MHC-I complexes using crystallography ^8, 75^ and NMR ^76, 77^.

A number of recent studies showed that TAPBPR could function as a peptide exchange catalyst ^76, 77, 80, 83^. Consistent with these findings, when TAPBPR is knocked out in cell lines, the number of unique peptides presented by MHC-I increases compared with cells expressing TAPBPR ^80^. Recent NMR studies provide considerable insights into the dynamics of peptide–MHC-I complexes in the presence and absence of TAPBPR and suggest mechanisms for functional activities of TAPBPR ^76, 77^. The murine MHC-I molecule H2-D ^d^ has been found to undergo specific localized conformational variations at sites of TAPBPR binding. Both peptides and TAPBPR individually mitigate the measured conformational dynamics of MHC-I. TAPBPR is suggested to function as a chaperone that allows for enhanced kinetic stability of peptide–MHC-I. TAPBPR forms a “latch” onto the MHC-I α _2–1_ region which is in dynamic equilibrium between open and closed conformations. High-affinity peptides that form stable interactions within the peptide-binding site can stabilize the closed latch conformation, thereby causing the dissociation of TAPBPR. This model suggests negative allosteric coupling between peptide–MHC-I and TAPBPR–MHC-I ^76, 77^. Furthermore, deep mutagenesis studies confirm that key TAPBPR binding sites are located within the α _2_ domain of the MHC-I peptide-binding site but that TAPBPR binding is generally tolerant to substitutions in the α _1_ domain ^77^. Thus, local folding in a nascent MHC-I molecule may be sufficient to induce TAPBPR binding as a chaperone and this function is suggested to have broad and multi-allele specificity. On the other hand, allele-dependent binding of TAPBPR to peptide-bound MHC-I has been found to be related to the distinct dynamic profiles of MHC-I allotypes. Peptide–MHC-I complexes that display high conformational dynamics at the sites of TAPBPR binding (defined by both the identity of bound peptide and the intrinsic features of individual heavy chain) are selectively recognized by TAPBPR and predicted to be subjected to more extensive TAPBPR-mediated editing ^77^. A recent study addressed the role of the TAPBPR helical loop called the scoop loop ^84^. The loop is proposed to be positioned to interact within the F pocket of the peptide-binding site. This placement of the scoop loop is suggested to generate competition for the incoming peptide substrates and allow the selective binding of only high-affinity substrates with MHC-I ^84^. This model remains somewhat controversial, as there are discrepancies in the placement of the loop between the two crystal structures ^8, 75, 85^.

A recent comprehensive analysis of TAPBPR binding to multiple human HLA-I allotypes revealed preferential binding of TAPBPR to HLA-A allotypes over HLA-B and HLA-C ^82^. There is an additional hierarchy among the HLA-A allotypes, and members of the A*02 and A*24 supertypes demonstrate preferential binding to TAPBPR. Interestingly, the addition of soluble TAPBPR to cells facilitates peptide exchange on the surface from selected HLA-I allotypes ^83^, which can be used to generate peptide–MHC-I libraries *in vitro* ^86^. TAPBPR binding preferences for a given allotype correlate with the ability of TAPBPR to mediate cell surface peptide exchange on the respective allotype ^82^. Residues H114 and Y116 in the F pocket of MHC-I have been found to be key determinants of TAPBPR binding. Additionally, amino acid residue M12, present on HLA-A*68:02, one of the strongest measured TAPBPR binders, has a strong influence on TAPBPR binding. H114 and Y116 are buried within the F pockets of HLA-I molecules forming contacts with C-termini of bound peptides, and their effects on TAPBPR binding are likely driven via an indirect influence on HLA-I residue dynamics, induced by peptide repertoire variations. Similarly, a conformational (rather than direct) influence is predicted for M12 ^82^.

Notably, HLA-B and HLA-C allotypes lack the H114/Y116 residue pattern. Introduction of H114/D116 amino acid residues in the F pockets of HLA-B induced TAPBPR binding and cell surface peptide exchange. As noted above, residues 114 and 116 have been previously shown to be important (but not the sole) determinants of HLA-B dependencies on tapasin ^47– 49, 57– 59^. For a small subset of HLA-I allotypes, the hierarchies of tapasin and TAPBPR binding are matched ^82^, suggesting similarities in chaperone requirements and preferences between TAPBPR and tapasin. Nonetheless, numerous HLA-B allotypes display very strong dependencies on tapasin for their cell surface expression whereas TAPBPR binding is poor for these HLA-B allotypes, highlighting important differences between the modes of tapasin and TAPBPR function. Given the structural relatedness of tapasin and TAPBPR ^8, 74– 77^, the molecular basis for such functional differences and the functional basis of the preferential activity of TAPBPR toward HLA-A remain to be elucidated. The functional differences between the two MHC-I–dedicated chaperones are likely driven by the weaker affinity of the tapasin–MHC-I complex compared with the TAPBPR–MHC-I complex, the incorporation of tapasin into the PLC, and subcellular localization differences. Overall, TAPBPR functions as a powerful peptide editor for several HLA-A molecules. Tapasin also functions as a peptide editor for a distinct group of alleles ^87– 89^, although its assembly-promoting (chaperone) function within the PLC may be dominant ^90^.

## Summary

Human MHC-I molecules are highly polymorphic with specific peptide motif preferences that are now being visualized in expanding numbers, which allow more accurate predictions of peptide repertoire sizes and antigenic epitopes. Spliced peptides, originating from distinct protein precursors, and post-translationally modified peptides are components of the peptide repertoires of MHC-I molecules. HLA-I molecules exhibit varying TAP and tapasin dependencies, and there are distinct influences of TAPBPR on HLA-I allotype assembly and surface expression. Several HLA-I molecules are able to bypass the conventional assembly route, which can confer selective immune advantages and be exploited for immunotherapy. The CD8-binding site of MHC-I is a common CD8 and chaperone interaction region, the conformation of which is sensitive to MHC-I peptide occupancy. The finding that CD8 preferentially engages peptide-deficient MHC molecules indicates the existence of common mechanisms of MHC-I conformational sensing by a cell surface receptor and ER chaperones and shows that MHC-I conformational sensing directly influences immunity.
